# Fibrillin-1 (FBN-1) a new marker of germ cell neoplasia in situ

**DOI:** 10.1186/s12885-016-2644-z

**Published:** 2016-08-04

**Authors:** Z. Cierna, M. Mego, I. Jurisica, K. Machalekova, M. Chovanec, V. Miskovska, D. Svetlovska, K. Kalavska, K. Rejlekova, K. Kajo, J. Mardiak, P. Babal

**Affiliations:** 1 Department of Pathology, Faculty of Medicine, Comenius University, Bratislava, Slovakia; 2 2nd Department of Oncology, Faculty of Medicine, Comenius University, Bratislava, Slovakia; 3 Translational Research Unit, Faculty of Medicine, Comenius University, Bratislava, Slovakia; 4 Princess Margaret Cancer Centre, University Health Network and University of Toronto, Toronto, Canada; 5 St. Elisabeth Cancer Institute, Bratislava, Slovakia; 6 1st Department of Oncology, Faculty of Medicine, Comenius University, Bratislava, Slovakia; 7 National Cancer Institute, Bratislava, Slovakia; 8 2nd Department of Oncology, Faculty of Medicine, National Cancer Institute, Comenius University, Klenova 1, 833 10 Bratislava, Slovak Republic

**Keywords:** Germ cell neoplasia in situ, Fibrillin-1, Testicular cancer

## Abstract

**Background:**

Germ cell neoplasia in situ (GCNIS), is preinvasive stage of testicular germ cell tumours (TGCTs). Fibrillins, which are integral components of microfibrils are suggested to be involved in cancer pathogenesis and maintenance of embryonic stem cells pluripotency. The aim of this study was to examine fibrillin-1 (FBN-1) expression in TGCTs patients.

**Methods:**

Surgical specimens from 203 patients with TGCTs were included into the translational study. FBN-1 expression was evaluated in the tumour tissue, in GCNIS and in adjacent non-neoplastic testicular tissue in all available cases. Tissue samples were processed by the tissue microarray method. FBN-1 was detected by immunohistochemistry using goat polyclonal antibody and the expression was evaluated by the multiplicative quickscore (QS).

**Results:**

The highest FBN-1 positivity was detected in GCNIS (mean QS = 11.30), with overexpression of FBN-1 (QS >9) in the majority (77.1 %) of cases. Expression of FBN-1 in all subtypes of TGCTs was significantly lower in comparison to expression in GCNIS (all *p* <0.001). Seminoma had significantly higher expression compared to EC, ChC and TER (all *p* <0.05), but not to YST (*p* = 0.84). In non-neoplastic testicular tissue the FBN-1 positivity was very low (mean QS = 0.02). Sensitivity, specificity, positive and negative predictive value of FBN-1 expression for diagnosis of GCNIS were 97.1, 98.8, 98.6 and 97.7 %.

**Conclusions:**

FBN-1 is overexpressed in TGCTs and especially in GCNIS when compared to non-neoplastic testicular tissue in patients with germ cell tumors and could be involved in germ cell neoplasia in situ development.

## Background

Testicular cancer accounts for approximately 1 % of all male tumours globally and represents the most common malignancy diagnosed among men between 15 and 44 years [1]. The worldwide incidence is 1.5 per 100 000 [2] with distinct age, country and race distribution; however, it has been increasing in recent decades [1, 3, 4]. Testicular germ cell tumours (TGCTs) represent 98 % of all testicular neoplasms. Germ cell neoplasia in situ (GCNIS) is a precursor lesion for invasive TGCTs of the adult testis [5] with the exceptions of prepubertal type teratomas which are rare in adults and spermatocytic tumours typical for elderly men and infantile germ cell tumours [6, 7].

GCNIS under term carcinoma in situ was first described in 1972 by Skakkebaek et al., in men who developed embryonal carcinoma of the testis, as atypical germ cells in seminiferous tubules suggested to represent a carcinoma in situ [8]. Germ cell neoplasia in situ is present adjacent to the invasive tumours in 72–98 % of the cases, in contralateral testis in 4.9–6.6 % of cases and sporadic finding in 0.4–0.8 % of the testes of otherwise healthy males [9]. If left untreated the risk of development of invasive testicular cancer is 70 % after 7 years [6].

GCNIS is defined as malignant germ cells confined to seminiferous tubules, which usually lack normal spermatogenesis [9]. Morphologically GCNIS comprises of germ cells with abundant vacuolated cytoplasm, large, irregular nuclei and prominent nucleoli located within the seminiferous tubules [10]. Atypical cells are basally arranged, interspersed with Sertoli cells [11]. Affected seminiferous tubules usually have thickened peritubular basement membrane [9]. In GCNIS detection, morphology and immunohistochemistry are commonly used to distinguish neoplastic cells from normal germ cells. Markers, which are expressed by GCNIS cells in varying intensity and percentage of cases include OCT3/4, NANOG, PLAP (placental alkaline phosphatase), AP2γ, MAGEA4 (melanoma antigen-encoding gene-A4), VASA, TSPY (the testis-specific protein, Y-encoded), CD117 (c-kit), M2A, 43-9F, TRA-1-60, TCL1 (T-cell leukemia/lymphoma 1), DDX3Y, podoplanin (D2-40) and SCF (stem cell factor) [10–17].

Emerging data suggest that fibrillins are involved in cancer pathogenesis [18–22]. Fibrillins are the primary components of microfibrils in the extracellular matrix of many elastic and non-elastic connective tissues [23]. Fibrillin-1 (FBN-1), fibrillin-2 (FBN-2) and fibrillin-3 (FBN-3) constitute the fibrillin family [24]. The majority of fibrillin molecules are multidomain cysteine-rich glycoproteins containing 43 calcium-binding epidermal growth factor-like domains and seven 8-cysteine-containing TB motifs [25–27]. FBN-1 and −2 are expressed in humans in most of tissues from 5th gestational week; however FBN-2 expression is reduced soon after birth, while FBN-1 remain expressed in adult tissues [24]. Fibrillins are mostly mentioned in connection with genetically determined diseases. Mutations in the gene for fibrillin-1 cause autosomal dominant disorder Marfan syndrome and other related diseases of connective tissue collectively named type-1 fibrillinopathies [28].

Recent data suggest that FBN-1 plays an important role in maintenance of pluripotency of embryonic stem cells [29]. Cancer cells in GCNIS as well as in TGCTs exhibit several similarities with embryonic stem cells; therefore in this translational study we investigated expression of FBN-1 in patients with TGCTs. FBN-1 expression was examined in all subtypes of TGCTs, in samples with germ cell neoplasia in situ and in adjacent non-neoplastic testicular tissue.

## Methods

### Study patients

Into this translational study (Protocol IZLO1, Chair: M. Mego), 203 patients with TGCTs treated from January 1999 to December 2013 in the National Cancer Institute of Slovakia and/or St. Elisabeth Cancer Institute, with available paraffin embedded tumour tissue specimen, were included. Patients with concurrent malignancy other than non-melanoma skin cancer in the previous 5 years were excluded. The Institutional Review Board approved this retrospective study and a waiver of consent form was granted.

### Tumour pathology

Pathology review was conducted at the Department of Pathology, Faculty of Medicine, Comenius University, by two pathologists (ZC and PB) associated with the study.

### Diagnosis and tissue samples

Tissue specimens were obtained before administration of systemic therapy. Tumour tissue, samples with germ cell neoplasia in situ and non-neoplastic testicular tissue were evaluated in all cases, when available. The TGCTs were classified according to World Health Organization criteria [10]. GCNIS was assessed based on morphological features and confirmed immunohistochemically with monoclonal antibody against OCT3/4. Normal testicular tissue from non-cancer patients was not available for analyses; therefore we used non-neoplastic tissue adjacent to testicular tumour for FBN-1 expression evaluation, as described in previous studies [30–32]. Non-neoplastic adjacent testicular tissue was available for FBN-1 expression evaluation in 85 patients. Non-neoplastic testicular tissue was categorized according to McLachlan et al. as follows: 1. Normal testicular biopsy; 2. Hypospermatogenesis; 3. Germ cell arrest; 4. Sertoli cell-only appearance (syndrome); 5. Seminiferous tubule hyalinization [33]. Out of these categories, FNB-1 expression was evaluated only in tissues with normal testicular biopsy, hypospermatogenesis or germ cell arrest.

### Tissue microarray construction

According to tumour histology, one or two representative tumour areas from each histological subtype (1–6 cores from each tumor) of germ cell tumour were identified on H&E sections. In case of mixed germ cell tumors, selected GCT histologies were sampled to isolate a specific histological pattern. Samples from non-neoplastic testicular tissue and germ cell neoplasia in situ were also marked, if present. Sections were matched to their corresponding wax blocks (the donor blocks), and 3-mm diameter cores of the tumour were removed from these donor blocks with the multipurpose sampling tool Harris Uni-Core (Sigma-Aldrich, Steinheim, Germany) and inserted into the recipient master block. The recipient block was cut into 4-μm sections that were transferred to coated slides.

### Immunohistochemical staining

Slides were deparaffinised and rehydrated in phosphate buffered saline solution (10 mM, pH 7.2). The tissue epitopes were demasked using the automated water bath heating process in Dako PT Link (Dako, Glostrup, Denmark); the slides were incubated in TRIS-EDTA retrieval solution (10 mM TRIS, 1 mM EDTA pH 9.0) at 98 °C for 20 min. The slides were subsequently incubated overnight at 4 °C with the primary goat polyclonal antibody against fibrillin-1 (Santa Cruz Biotechnology, fibrillin-1 (N-19): sc-7541) diluted 1:100 in Dako REAL antibody diluent (Dako, Glostrup, Denmark) and immunostained using anti-goat universal immuno-peroxidase polymer (Histofine, Nichirei Biosciences, Japan) for 30 min at room temperature, according to the manufacturer’s instructions. Color reaction was developed with diaminobenzidine substrate-chromogen solution (DAB, Dako, Glostrup, Denmark) for 5 min. Finally, the slides were counterstained with haematoxylin. Samples of human placenta tissue served as positive control and no staining in slides with omission of the primary antibody served as negative control.

### Immunohistochemical stain scoring

Tumour cores were independently assessed by two observers (ZC and PB) who were blinded to clinicopathological data, as image analysis for cytoplasmic staining is fraught with technical difficulties in controlling the parameters to do the study. In cases of disagreement, the result was reached by consensus. Fibrillin-1 expression was scored by the multiplicative quickscore method (QS), which accounts for both the extent of cell staining as well as staining intensity. Briefly, the portion of positive cells was estimated and given a score on a scale from 1 to 6 (1 = 1–4 %; 2 = 5–19 %; 3 = 20–39 %; 4 = 40–59 %; 5 = 60–79 %; and 6 = 80–100 %). The average intensity of the positive staining was given a score from 0 to 3 (0 = no staining; 1 = weak; 2 = intermediate; and 3 = strong staining). QS was then calculated by multiplying the percentage score by the intensity score, to yield a minimum value of zero and a maximum value of 18. Based on the QS, cytoplasmic FBN-1 expression was graded as low (0–9) or high (10–18) as we described previously [31]. Identical scoring system was applied for mixed vs. pure GCTs, and each histological subtype in mixed GCTs was score separately.

### Statistics

The distribution of the FBN-1 QS was significantly different from the normal distribution (Shapiro–Wilk test); therefore we used non-parametric tests for analyses. Analyses of differences in distributions of FBN-1 expression between the two groups of patients were performed using the Mann–Whitney U test, while Fisher’s exact test or the χ2 test when appropriate were used, when FBN-1 expression was categorized as ‘low’ or ‘high’ according to the aforementioned criteria. All reported p values were two sided. For all statistical analyses, a *p* value <0.05 was considered as significant. Statistical analyses were performed using NCSS 2007 software (Hintze J, 2007, Kaysville, Utah, USA). Graphs were generated using GraphPad Prism Software, version 6, CA, USA).

## Results

Tumour specimens from 203 patients before administration of systemic therapy included 77 pure seminomas (SEM), 44 embryonal carcinomas (EC), nine yolk sac tumours (YST), one choriocarcinoma (ChC) and eight teratomas (TER). The 64 tumours were mixed germ cell tumours (Table 1). We identified GCNIS adjacent to testicular tumour in 70 tumour specimens as well. In total, we evaluated 350 tumour specimen and 85 adjacent non-neoplastic testicular tissues in 203 patients (Table 2).

**Table 1:** Composition of mixed TGCTs (*N* = 64)

Histological subtype					Number of patients
EC				TER	21
EC	SEM				12
EC			ChC	TER	6
		YST		TER	6
EC			ChC		3
EC		YST			2
EC		YST		TER	2
	SEM		ChC		2
	SEM			TER	2
			ChC	TER	2
EC	SEM		ChC		1
EC	SEM		ChC	TER	1
EC	SEM			TER	1
EC	SEM	YST			1
	SEM	YST			1
		YST	ChC		1

*Abbreviations*: *EC* embryonal carcinoma, *SEM* seminoma, *YST* yolk sac tumour, *ChC* choriocarcinoma, *TER* teratoma

**Table 2:** Fibrilin-1 expression in TGCTs

		Fibrilin expression						
Histological subtype	*N*	Mean QS	Standard error of the mean	Median QS	*p*-value^a^	Low^b^	High^c^	*p*-value^a^
						*N* (%)	*N* (%)	
GCNIS	70	11.30	0.54	12	NA	16 (22.9)	54 (77.1)	NA
SEM	98	6.11	0.49	3	<0.001	73 (74.5)	25 (25.5)	<0.001
EC	94	4.53	0.37	4	<0.001	83 (88.3)	11 (11.7)	<0.001
YST	22	5.59	0.71	6	<0.001	18 (81.8)	4 (18.2)	<0.001
TER	49	3.37	0.49	3	<0.001	46 (93.9)	3 (6.1)	<0.001
ChC	17	0.41	0.23	0	<0.001	17 (100.0)	0 (0.0)	<0.001
Non-neoplastic testis	85	0.02	0.02	0	<0.001	85 (100.0)	0 (0.0)	<0.001

Note that 64 tumours were mixed germ cell tumours (see text)

*Abbreviations*: *GCNIS* germ cell neoplasia in situ, *NA* not applicable, *QS* multiplicative quickscore

^a^Compared to GCNIS

^b^QS 0–9

^c^QS 10–18

Fibrillin-1 expression was detected in 85 (86.7 %) of seminomas, 78 (83.0 %) of embryonal carcinomas, 21 (95.5 %) of yolk sac tumors, 3 (17.6 %) of choriocarcinomas, 35 (71.4 %) of teratomas, 68 (97.1 %) of GCNIS and 1 (1.2 %) non-neoplastic tissue. Fibrillin-1 expression according to multiplicative quickscore in different histological subtypes of germ cell tumours, in GCNIS and non-neoplastic testicular tissue is showed in Table 2. The highest FBN-1 positivity was found in GCNIS (mean QS ± SEM = 11.30 ± 0.54), with overexpression of FBN-1 (QS >9) in the majority (77.1 %) of cases.

**Fig. 1:**
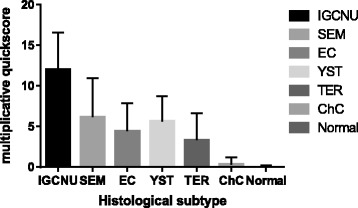
FBN-1 expression in different TGCTs histological subtypes

**Fig. 2:**
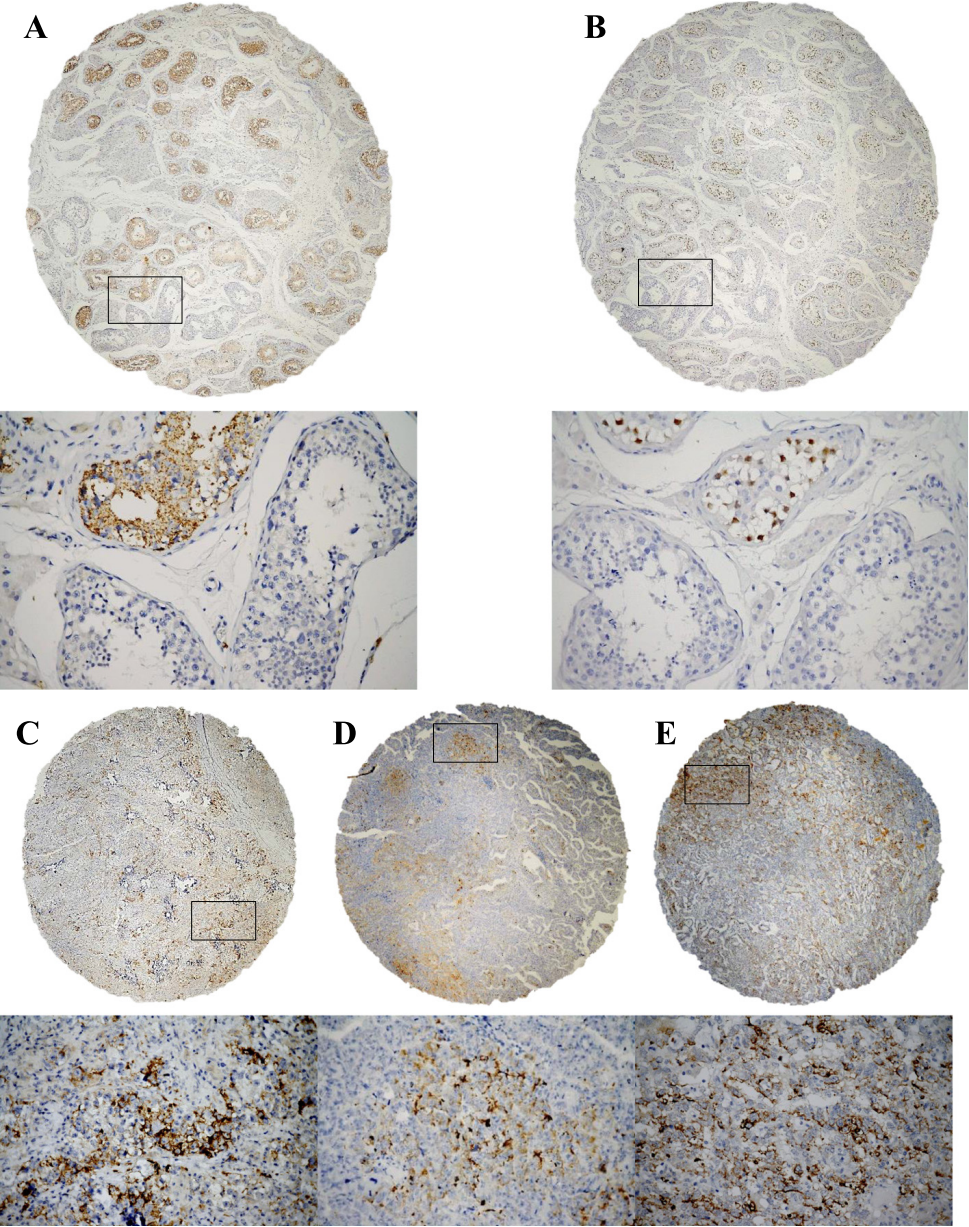
Immunohistochemical detection of fibrillin-1 (FBN-1) and OCT3/4 expression in testicular tissues. **a** Germ cell neoplasia in situ (GCNIS), showed constant and strong cytoplasmic granular FBN-1 positivity (*brown colour*) with negativity in normal seminiferous tubules with spermiogenesis; (**b**) OCT3/4 expression in the same sample as in (**a**) with strong nuclear positivity in GCNIS (*brown colour*) and negativity in normal tubules. **c** Seminoma with focal strong cytoplasmic FBN-1 positivity; (**d**) Embryonal carcinoma with focal moderate to strong cytoplasmic FBN-1 positivity; (**e**) Yolk sac tumour with focal strong cytoplasmic FBN-1 positivity. Original magnification ×40/×400

Expression of FBN-1 in all subtypes of TGCTs (seminoma, embryonal carcinoma, yolk sac tumour, teratoma, choriocarcinoma) was significantly lower in comparison to the expression in GCNIS (Table 2, Fig. 1). Most of the testicular tumours had predominance of cases with low QS <9: seminoma (74.5 %), embryonal carcinoma (88.3 %), yolk sac tumour (81.8 %), teratoma (93.9 %) and all choriocarcinomas (100 %) (Fig. 2). In non-neoplastic testicular tissue only very low FBN-1 positivity was detected (mean QS = 0.02). Only in 1 (<1 %) case of non-neoplastic tissue germ cells exhibited weak FBN-1 positivity but it was only focal in a small number of tubules, all other cases were negative for FNB1 (Fig. 2). There was no difference in FBN-1 expression between pure or mixed germ cell tumours (*p* = 0.45). Seminoma had significantly higher expression when compared to EC, ChC and TER (all *p* <0.05), but not to YST (*p* = 0.84).

Only two cases (2.9 %) of GCNIS were FBN-1 negative, median grade of FBN-1 expression in GCNIS was 12, while median of cell positivity was 100 %. Sensitivity, specificity, positive and negative predictive value of FBN-1 expression for diagnosis of GCNIS were 97.1, 98.8, 98.6 and 97.7 %. Moreover, sensitivity, specificity, positive and negative predictive value of FBN-1 overexpression (QS >9) for diagnosis of GCNIS were 77.1, 100.0, 100.0 and 84.2 %.

## Discussion

GCNIS represents the preinvasive stage of TGCTs of adults except for spermatocytic tumours and prepubertal type teratomas which are rare in adults. Germ cell neoplasia in situ cells are believed to be germ cells that have failed to undergo normal maturation during fetal or early postnatal life [12]. Physiologically during the intrauterine period, primordial germ cells (PGC) transform to gonocytes. PGC and gonocytes undergo DNA demethylation, which allows development of sex-specific germ cell lineages and these cells typically express markers of pluripotency PLAP, NANOG, OCT3/4, c-kit and AP2γ [9].

In our study, we found significantly higher expression of FBN-1 protein in GCNIS in comparison to non-neoplastic testicular tissue as well as to all histological subtypes of TGCTs. The appearance of FBN-1 expression in germ cells in the stage of GCNIS can be an early event in the process of malignant transformation. Our data suggest, that progression of GCNIS to overt germ cell tumour is connected with a decrease of FBN-1 production. Among TGCTs, the highest expression was observed in seminomas and the lowest in choriocarcinoma, suggesting a decrease of FBN-1 expression with the process of differentiation. In vitro studies indicated a significant role of FBN-1 for embryonic stem cells (ESCs). FBN-1 supported growth, self-renewal, attachment and maintenance of pluripotent human embryonic stem cells [29]. Due to similarities between ESCs and GCNIS, we suppose, that FBN-1 is involved in similar processes in GCNIS as in ESCs [29] and based on our results we suggest, that GCNIS cells start to produce FBN-1 in order to ensure growth and self-renewal of cancer cells. With further growth and differentiation of cancer cells, the importance of FBN-1 for attachment and self-renewal probably decreases, which could lead to the decreased expression of FBN-1 in the invasive TGCTs. Similarly, during the differentiation, the germ cells gradually lose expression of other markers associated with GCNIS including NANOG, PLAP, and OCT3/4 (POU5F1), and partially lose expression of KIT and SALL4, (reviewed by [34]), therefore observed FBN-1 expression in TGCTs is consistent with expression of these markers.

In our previous study we observed that PARP1 (poly(ADP-ribose)polymerase1) expression in TGCTs had similar expression pattern as FBN-1 [31]. PARP1 positivity was the highest in GCNIS and among germ cell tumours its expression was higher in less differentiated subtypes of tumours, seminoma and embryonal carcinoma, with decreased positivity in more differentiated subtypes. PARP1 regulates FBN1 gene expression through Sp1 [35] and these data suggest, that increased PARP expression in neoplastic cells in germ cell tumours can lead to increased FBN1 gene expression. Another study revealed, that FBN-1 expression is stimulated by Aurora A, a serine/treonine kinase, and is inhibited by tumour suppressor gene BRCA2 [36]. Previous studies showed increased expression of Aurora kinase proteins in GCNIS, thus further supporting regulation of FBN-1 expression by Aurora kinases [37, 38].

Fibrillins, in particular FBN-1 and −2 are integral components of microfibrils, which provide strength and elasticity to the matrix. Moreover, they are important for controlling the growth and differentiation of the cells they surround, through interaction via integrins and growth factors. They are involved in regulation of members of the TGF-β superfamily [39] through which they can affect several processes involved in cancer including induction of epithelial-mesenchymal transition (EMT) [40, 41], or regulation of matrix metalloproteinases expression [42]. While mutations in the gene for fibrillin-1 cause Marfan syndrome, there is limited data of association between Marfan syndrome and risk of testicular germ cell tumors [43].

## Conclusion

In conclusion, in this translational study we showed for the first time that FBN-1 is overexpressed in TGCTs and especially in GCNIS when compared to non-neoplastic testicular tissue in patients with germ cell tumors. Thus FBN-1 could be a factor involved in germ cell neoplasia in situ development. Our data provide new insight into tumorigenesis of TGCTs; however, further research is needed to validate the role of FBN-1 in TGCTs pathogenesis and to evaluate FBN-1 as a possible new therapeutic target in TGCTs.

## Abbreviations

EC, embryonal carcinomas; EMT, epithelial-mesenchymal transition; ESCs, embryonic stem cells; FBN-1, fibrillin-1; FBN-2, fibrillin-2; FBN-3, fibrillin-3; GCNIS, germ cell neoplasia in situ; ChC, choriocarcinoma; MAGEA4, melanoma antigen-encoding gene-A4; NA, not applicable; PARP1, poly(ADP-ribose)polymerase1; PGC, primordial germ cells; PLAP, placental alkaline phosphatase; QS, quickscore; SCF, stem cell factor; SEM, seminomas; TCL1, T-cell leukemia/lymphoma 1; TER, teratomas; TGCTs, testicular germ cell tumours; TSPY, the testis-specific protein, Y-encoded; YST, yolk sac tumours
